# Efficacy of nano-silver small intestine submucosa repair of osteochondral defect in rabbit model by the AMPK-mTOR-ULK1 pathway

**DOI:** 10.1186/s41065-026-00635-4

**Published:** 2026-01-19

**Authors:** Heng-Shu Wang, Chong Zhang

**Affiliations:** 1 Department of Pathology, The Second Hospital of Hebei Medical University, No. 215 Heping West Road, Xinhua District, Hebei Province 050000 Shijiazhuang, China; 2 Department of Orthopaedic Surgery, Traditional Chinese Medicine Hospital of Hebei Province, No. 389 Zhongshan East Road, Chang’an District, Shijiazhuang, Hebei Province 050011 China

**Keywords:** Growth factors, Nano-silver, Osteochondral defect, Small intestine submucosa, Tissue regeneration

## Abstract

**Objective:**

This study evaluated the regenerative potential of nano-silver small intestine submucosa (NSSIS) scaffolds with a 4-D porous structure for repairing osteochondral defects in rabbit knee joints.

**Methods:**

NSSIS scaffolds were prepared using nanosilver, fresh pig-derived small intestinal submucosa, and chondrocytes. Biocompatibility was assessed by methyl thiazolyl tetrazolium (MTT) assays measuring bone marrow stromal cell (BMSC) proliferation at 24, 48, and 72 h. A rabbit model of intercondylar groove cartilage defects was established and randomized into three groups (*n* = 12 each): NSSIS, NSSIS + BMSCs, and a control group. Scaffold morphology and cell growth were evaluated in vitro using H&E staining after 24 h. Following implantation, cartilage repair was assessed at 24, 48, and 72 h using ICRS macroscopic scoring and histological staining (H&E, Safranin O-fast green, toluidine blue). After 12 weeks, ELISA measured growth factor expression (PDGF, VEGF, TGF-β, IGF-1, FGF, EGF), and qRT-PCR and Western blotting assessed autophagy-related gene and protein expression (AMPK, ULK1, mTOR, and Beclin-1).

**Results:**

Both NSSIS groups demonstrated significantly greater BMSC ingrowth compared with controls, with the NSSIS + BMSCs group exhibiting the most robust repair. This group showed significantly elevated growth factor expression at 12 weeks (*p* < 0.05), downregulation of AMPK, ULK1, and Beclin-1, and upregulation of mTOR (*p* < 0.01). Histological analysis revealed enhanced chondrocyte formation, thicker cartilage layers, increased chondroblast proliferation, and abundant extracellular matrix deposition in the NSSIS + BMSCs group, whereas the NSSIS-only group showed less cellular and collagen development.

**Conclusions:**

NSSIS scaffolds demonstrate good biocompatibility and promote BMSC ingrowth, chondrocyte development, and osteochondral repair. The addition of BMSCs further enhances these effects by facilitating in situ chondrogenic differentiation, stimulating BMSC and chondrocyte migration, and initiating tissue regeneration. These findings highlight the potential of NSSIS, particularly when combined with BMSCs, as a promising biomaterial for cartilage and subchondral bone repair, with potential clinical applications in regenerative medicine.

## Introduction

Osteochondral injuries in the knee joint are commonly encountered in clinical practice; however, the limited regenerative capacity of osteochondral tissue poses a critical challenge for self-repair following damage. Current management strategies for focal chondral defects include a range of surgical techniques such as microfracture, autologous chondrocyte implantation, and osteochondral grafting, each with varying degrees of success depending on lesion characteristics [1, 2]. Tissue engineering has emerged as a promising approach for addressing osteochondral defects [3–6]. Small intestine submucosa (SIS), a decellularized collagen matrix, shows strong osteoinductive and osteoconductive potential for bone and cartilage repair due to its bioactive factors and scaffold properties [7, 8]. Widely used in tissue repair, SIS supports regeneration with good biocompatibility, Becher claimed that although contraction post-repair remains a limitation [9]. 

Nowadays, nanoparticles have attracted great attention due to their potential biomedical applications, such as iron oxide magnetic nanoparticles (IONPs) [10], activated carbon-coated magnetic nanocomposite [11]. In order to improve the utilization rate of artificial materials in China, this experiment integrates the functions of tissue biological activity and nano silver antibacterial stent, and gradually develops from a single to a complex new type of biological nano silver small intestinal submucosa complex with good histocompatibility and low rejection reaction. Historians have used it to repair abdominal wall defects or abdominal wall hernia, spinal dura repair and other major diseases. Recently, Bi X et al. have used small intestinal submucosa gel coating materials to achieve spinal dura repair [12]. Nano-silver small intestine submucosa (NSSIS) enhances the regenerative capacity of SIS by promoting scaffold ingrowth and stimulating increased growth factor release [13, 14]. Enriched with fibrin, NSSIS contains key cytokines and growth factors such as PDGF, TGF-β, IGF, VEGF, EGF, and FGF, which synergistically enhance cell proliferation, proteoglycan and type II collagen synthesis, and tissue regeneration [15], Its porous, loose clot structure retains high cellular content, including leukocytes and platelets, even in distal regions, maintaining biological activity. NSSIS has been shown to release higher levels of growth factors than platelet-rich plasma (PRP) or standard SIS [16], exerting strong mitogenic effects on chondrocytes and promoting cell differentiation [17, 18]. Furthermore, NSSIS significantly enhances cell migration and effectively recruits mesenchymal stem cells (MSCs) from bone marrow, gingiva, neural tissue, and other sources. While SIS already demonstrates chondrocyte recruitment [19], NSSIS amplifies this property, positioning it as a promising biomaterial for scaffold applications in osteochondral tissue engineering.

Currently, research on the use of SIS and NSSIS in bone defect repair has achieved notable progress; [20] however, their application in osteochondral defect repair remains relatively underexplored [21]. Despite these advances, bioprinting NSSIS exhibits stronger ability in inducing cartilage differentiation due to its looser structure and significantly higher levels of cytokines and growth factor components in tissues, which maintain high activity for preservation and preservation [22]. In this experiment, following the establishment of the model, NSSIS was implanted to assess its effects on the biocompatibility, proliferation, chondrogenic and osteogenic differentiation capabilities, and migration capacity of bone marrow mesenchymal stem cells (BMSCs). Thus, the objective of this study aimed to analyze the role of NSSIS repairing osteochondral defects in rabbit knee joints to evaluate its potential application in cartilage regeneration. After the transplantation of NSSIS + BMSCs composite material into cartilage defects, a reliable biological bonding interface can be reformed with the cartilage bed, achieving the reconstruction of transparent articular cartilage surface. Biopolymer materials can promote cartilage regeneration and repair, and have clinical application value in the field of regenerative medicine.

## Materials and methods

### Experimental animals and main reagents and instruments

All of the 36 healthy male New Zealand white rabbits (12 months old, 2.5–3.0 kg) were selected due to their robust physique and common use in bone defect models, with joint cartilage similar to humans in chondrocyte size and a higher capacity for repair [23]. The animals were purchased from the Experimental Animal College of Hebei Medical University in Chang’an District, Shijiazhuang City, China. They were kept in the Animal Laboratory of the Second Hospital of Hebei Medical University (Animal Production License Number: SCXK (Ji) 2022-001).

Methyl thiazolyl tetrazolium (MTT) (Amresco, USA); 10% neutral formalin fixative (Shanghai Yichi Biotechnology Co., Ltd., China); dimethyl sulfoxide (DMSO) (Sigma, USA); enzyme-linked immunosorbent assay (ELISA) kits (Shanghai Enzyme-linked Biotechnology Co., Ltd., China); live/dead cell staining kit (ScienCell, USA); and QuantiTect SYBR Green PCR kit (Qiagen, Germany). Centrifuge (TR-18plus; Jiangsu Trausim Medical Instrument Co., Ltd., China); iMark microplate reader (Bio-Rad, USA); NanoDrop 2000 spectrophotometer (Thermo Scientific, USA); absorbance microplate reader (ELX808; Bio-Tek, USA); Olympus optical microscope (BX51; Olympus, Japan); inverted fluorescence microscope (DMI12000B; Leica, Germany); 7900HT Fast Real-Time Fluorescent Quantitative PCR System (ABI, USA); and Transwell 24-well cell culture chamber (Corning, USA). Single nano silver particles with a diameter of 20 nm (model: AGS5000-PP/AGS-DMB5000-PP/AGS-PP, Shanghai Huzheng Industrial Co., Ltd. China).

### Preparation of 4-D porous structure NSSIS

In this study, the latest clinical and experimental literature was systematically retrieved from multiple databases, including CNKI, Web of Science & Science Citation Index, Medline, Embase, HighWire, CBM, and Cochrane Library. Searches were conducted using keywords and title terms such as “nano-silver,” “small intestine submucosa,” “osteochondral defect,” “growth factors,” and “tissue regeneration,” with Boolean operators (AND, OR, NOT) to refine results. Wildcards (*) and truncation were used to capture variations of key terms. Literature was selected from the most recent 1–3 years to ensure up-to-date evidence, while classic studies older than three years were also reviewed for foundational context. Search strategies included stepwise filtering through titles, abstracts, and key phrases to identify relevant studies, and the experimental design was informed by the evidence derived from this literature.

Take fresh pig small intestine of 8–10 cm, manually and mechanically separate the mucosal layer and muscular layer of the small intestine, wash it, soak it in sterile deionized water, and shake it on a shaker at 200r/min for 24 h; Soak in a 2% peracetic acid solution for 2 h, wash 8 times with sterile deionized water for 25 min each time, soak in a solution containing 0.1% peracetic acid and 20% anhydrous ethanol for 2 h, rinse 8 times with sterile deionized water for 25 min each time; Finally, store in sterile deionized water containing 1% streptomycin and store at 4 °C. Soak the SIS material in 100% fetal bovine serum before surgery and incubate it in a 37 °C incubator for 24 h. Place the prepared submucosal layer of pig small intestine into a prepared 40 µg/mL monomeric nanosilver solution, shake it on a shaker for 24–48 h to obtain NSSIS composite bioremediation material, and store it at 4 °C for future use.

Rabbit BMSCs were isolated using flow cytometry. Most red blood cells, adipocytes, and platelets were effectively removed using a lymphocyte separation medium with a density of 1.077, yielding a relatively pure BMSC population. After 48 to 72 h of careful culture, the BMSCs exhibited uniform morphology, clustered together, and demonstrated adherent growth. Extraction and cultivation of rabbit primary BMSCs → passaging to three generations → initial density of 3 × 10^5^ cell/cm^2^ for osteogenic induction → lipid induction → induction of cartilage formation → collect culture medium, stored at −80 ℃, implantment density not less than 2 × 10^6^ cell/m. The simplified procedure included the following steps: isolation → primary culture → expansion → collection. Prior to implantation, NSSIS group of NSSIS material was incubated in 100% fetal bovine serum (FBS) at 37 °C for 24 h. The material of NSSIS + BMSCs group was incubated in 100% fetal bovine serum (FBS) at 37 °C and PAA (Polyacrylic Acid) in order for 24 h respectively. The carboxyl group of polyacrylic acid increases biocompatibility of enhancement utilization. The control group was treated as a sham surgery group. The joint capsule was incised to observe the growth of the intercondylar fossa defect area without any implantation and then the suture incision was closed again.

Isolation and Culture of Rabbit Chondrocytes: To prevent rejection reactions, reduce peroxidation and improve the hollowing effect of regenerated tissue, the study applied Methylprednisolone sodium succinate (MPss) before euthanizing animals. The drug was directly injected into the subarachnoid space via intrathecal injection, allowing it to quickly diffuse into the cerebrospinal fluid and reach an effective blood concentration of 6.0 mg/kg, once daily for 3 to 5 days.

Take the cartilage at the apex of the posterior cartilage edge of the lateral wall of the intercondylar fossa, specifically the cartilage area with a bone tunnel diameter of 7.5 mm located at the center point of the 140° arch arc on the inner side of the lateral condyle, 2 mm behind the intercondylar crest and the intercondylar fossa crest on the inner and outer platforms of the tibial tunnel. Rabbit chondrocytes were isolated by euthanizing the rabbits with excess sodium pentobarbital anesthesia. Under sterile conditions, cartilage tissue was excised from the surface of the rabbit knee joints and minced. The minced tissue was digested in a 0.25% type II collagenase solution at 37 °C for 3 h. After digestion, the tissue suspension was pipetted for 1 min, allowed to settle, and the supernatant was collected and centrifuged (180×g, 5 min). The resulting cell pellet was resuspended in DMEM containing 10% FBS and 1% penicillin-streptomycin.

The cells were cultured under standard conditions (37 °C, 5% CO_2_, and 100% humidity) in an incubator until passage.

### Collection and preparation of NSSIS + BMSCs for histological analysis

Specimen Fixation and Section Preparation: Chemically synthesized NSSIS and nano-silver composites were fixed in 10% neutral formalin buffer for 24 h. The specimen was dehydrated through an ethanol gradient and embedded in paraffin. We obtained rabbit chondrocytes, collected the supernatant from the culture medium, and added NSSIS to detect their growth.

The tissue sections were cut to a thickness of 5 μm. Hematoxylin and eosin (H&E) staining were performed. The stained tissue sections were observed under a microscope and images were captured for further analysis.

### Scanning electron microscopy (SEM) and histological observation of NSSIS + BMSCs structure

The interface bonding strength between NSSIS + BMSCs tissue, namely nano silver and SIS & BMSCs substrate, was tested by an electronic universal tensile strength tester (S2 series, Shanghai Sturma) for tensile compression bending before implantation. Overlapping shear curves and adhesive strength measurements were taken, and the average size was approximately 150 mm² and 200–250 N (Stress testing is not the focus of this article, so it will not be discussed further).

After effective decellularization of nano silver small intestinal submucosal scaffold cells in vitro, they were placed in a quantitative solution of type II collagen and aggrecan for optical heat treatment, and then decellularized at an appropriate seeding density to obtain a dense endothelial cell layer. Preparation of sample histological analysis using scanning electron microscopy (SEM): The prepared NSSIS + BMSCs was fixed in 2.5% glutaraldehyde at room temperature for 2 h, and then washed with phosphate buffered saline (PBS). The sample was dehydrated through a series of graded ethanol concentrations. NSSIS + BMSCs specimens were fixed in 10% neutral formalin, embedded in paraffin, and vertically sliced to a thickness of 5 µ m.

In order to facilitate the adhesion and growth of chondrocytes and BMSCs and induce differentiation into chondrocytes, the samples were examined under SEM at 0 kV and 200x magnification, and the sections were stained with H&E analyzed.

### Rabbit knee joint osteochondral defect modeling, grouping, and joint scoring

Osteochondral Defect Modeling Procedure: Rabbits were anesthetized via intravenous injection of 3% sodium pentobarbital (1 mL/kg) into the ear vein. A lateral patellar incision was made, and the patella was dislocated to expose the knee joint. A cylindrical full-thickness osteochondral defect measuring 5 mm in diameter and 3 mm in depth was created in the patellar groove using a surgical drill.

Both NSSIS + BMSCs and NSSIS brackets are prepared to be made into cylindrical shapes, matching the defect size area with a mold diameter of about 5 mm and a depth of about 3 mm. Experimental Grouping: A total of 36 successful modeling rabbits were randomly divided into three experimental groups: NSSIS group (*n* = 12): implanted with NSSIS; NSSIS + BMSCs group (*n* = 12) implanted NSSIS + rabbit BMSCs; the control group (*n* = 12) without implanting any material. MPss intrathecal injection of 3.0 mg/kg, qd, for 5 days.

Postoperative Assessment: After implantation of the organism into the rabbit model, at 24, 48, and 72 h, the rabbits were euthanized for specimen collection. Macroscopic evaluation of the joint surface was conducted using the International Cartilage Repair Society (ICRS) scoring system [24, 25]. The scoring system assesses four parameters: the extent of defect filling, the color match of the repaired cartilage, the integration with surrounding cartilage, and the smoothness of the cartilage surface. Scores range is from 0 to 16, with higher scores indicating better cartilage repair outcomes.

### Detection of growth factors in NSSIS using ELISA after 12 weeks of implantation

Preparation and Analysis: In order to maintain the activity of NSSIS and NSSIS + BMSCs specimens and protect the structure and function of biomolecules in experimental samples, they were incubated and stored in 20 mL PBS at 37° C.

Collect the supernatant of specimens and evaluate the release of growth factors (PDGF, VEGF, TGF-β, IGF, FGF, and EGF) using method of ELISA. The ELISA procedure was as follows: A total of 40 µL of the assay diluent and 10 µL of the collected supernatant were placed in 96-well plates pre-coated with specific antibodies and incubated at 37 °C for 30 min. The wells were washed 5 times with a wash buffer. A horseradish peroxidase-conjugated antibody solution was added to the wells and incubated for 30 min. Substrate solution was then added, and the plate was incubated in the dark for 15 min. Finally, 50 µL of stop solution was added to terminate the enzyme reaction. The absorbance was measured at 450 nm using an iMark microplate reader. All samples were tested in triplicate to ensure accuracy.

### Observation of rabbit NSSIS + BMSCs histology

Histological Analysis: After implantation of the construct into the rabbit model, at 24, 48, and 72 h, the implanted specimens were removed, immediately fixed in 10% neutral formalin, and then decalcified. The specimens were embedded in paraffin and sectioned vertically into 5-µm-thick slices. Histological staining was performed using H&E, Safranin O-fast and green toluidine blue staining. Toluidine Blue and Safranin O staining bind to the negatively charged proteoglycans in the cartilage, allowing evaluation of the cartilage matrix distribution, the healing interface between the implanted NSSIS + BMSCs and the host cartilage, the integration of newly formed cartilage with the subchondral bone, and in vivo degradation of NSSIS. Histological examinations were independently performed by two experienced pathologists who were blinded to the experimental groups. Quantitative histological analysis was conducted using ImageJ software to measure parameters such as cartilage thickness, Safranin O–positive area fraction, and the percentage of newly formed cartilage tissue at the defect site.

### Detection of the effect of NSSIS on gene expression in rabbit BMSCs using qRT-PCR after 12 weeks of implantation

Experimental Procedure: Cell Culture and Treatment: Rabbit BMSCs were cultured with NSSIS in DMEM containing 10% FBS and 1% penicillin-streptomycin for 7 days. The control group was cultured in the same medium without NSSIS.

RNA Extraction and cDNA Synthesis: Total RNA was extracted from the cultured cells. RNA concentration and purity were determined using a NanoDrop2000 spectrophotometer, and 0.5 µg of RNA was used to synthesize cDNA using a commercial kit (Promega, USA).

Gene Expression Analysis: The expression of adenosine 5-monophosphate (AMP)-activated protein kinase (AMPK), Unc-51-like kinase 1 (ULK1), mammalian target of rapamycin (mTOR), and Beclin-1 protein (Beclin-1) receptors in rabbit cartilage was assessed.

qRT-PCR Setup: PCR reactions were performed using a 7900HT Fast Real-Time PCR system with antibodies for AMPK, ULK1, mTOR, Beclin-1 protein (Shanghai United Imaging Healthcare Co., Ltd, China, Catalog Number: LC1010-3). The QuantiTect SYBR Green PCR Kit was used to quantify the gene transcription levels of type II collagen, aggrecan, ALP, and OCN. Primers specific for each target gene were designed, as listed in Table 1. After DNA denaturation and dissociation, the annealing temperature for complementary sequence pairing and binding was lowered to 55℃, and the length of the extended amplicon was 146 bp. Real-time PCR reactions were carried out at 94 °C for 15 s, followed by a 30-second extension step at 125 °C.

**Table 1:** Primer sequences for qRT-PCR

Gene	Sequence	
	Forward (5’ → 3’)	Reverse (5’ → 3’)
AMPK	TCCTGTGCGACGACATAAT	CCTTTGGTCCTGGTTTCC
ULK1	CCCGAGAATCAAATGG	TAGTTGGGCAGCGAGA
mTOR	CGTGGCAACTCCATCTT	AGGGTTTCTTGTCCGTGT
Beclin-1	ACTCTTGTCGCCCTGCTG	TCGCTGCCCTCCCTCT

Normalization and Data Analysis: The transcription levels were normalized to β-actin as the internal reference. Relative expression was calculated using the 2 − ΔΔCt formula.

### Western blot analysis of the expression of AMPK, ULK1, mTOR and Beclin-1 related proteins in articular chondrocytes

Chondrocytes in each group were collected after 12 weeks of implantation and the total protein of chondrocytes was extracted. The detection process was as follows: tissue protein extraction → determination of protein concentration by BCA method → protein denaturation → preparation of electrophoresis gel → electrophoresis separation → Western blot color development. ECL color development system was closed with 5% skim milk powder. The dilution ratio of AMPK, ULK1, mTOR and Beclin-1 antibodies (orb167533) was 1:1500, and the anti-incubation solution was incubated overnight at 4 °C. TBST film washing 5 times, 10 min/time; The incubation band corresponding to 1:4000 was added for 1 h, and the reaction was conducted at room temperature for two hours. TBST film washing 5 times, 15 min/time; ECL solution was used for exposure development. The percentage ratio of the target band to the integral absorbance value of the internal reference band was used to express the expression level of each detection-related protein.

### Statistical analysis

SPSS 24.0 statistical software was used to process and analyze the data. Data conforming to a normal distribution were represented as the mean ± standard deviation (x̄ ± s). For data with homogeneity of variance, one-way analysis of variance (ANOVA) was used to compare the means among groups, followed by the Least Significance Difference (LSD) test for pairwise comparisons. For data with heterogeneity of variance or non-normal distribution, the Kruskal–Wallis H test was applied. A *p*-value of < 0.05 and < 0.01 indicated statistical significance.

## Results

### ICRS score (Primary outcome)

**Fig. 1:**
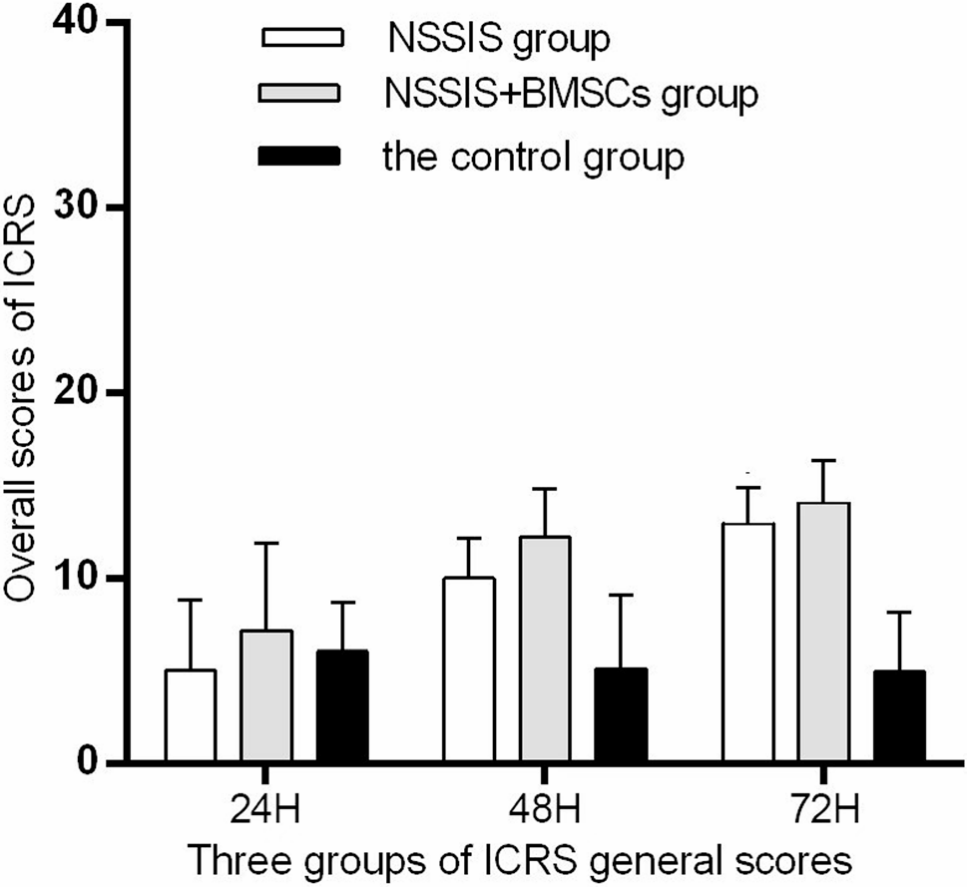
Detection of cytotoxicity and proliferative ability of NSSIS

All animals recovered well without symptoms of infection. After specimen removal, the articular surface appeared shiny, resembling normal cartilage. The defect was largely repaired by translucent tissue, and its boundary gradually blurred. In the NSSIS + BMSCs group, the boundary was almost indistinguishable from surrounding normal cartilage, and the defect was nearly restored. In the NSSIS group, cartilage repair was evident, while in the control group the defect was only slightly covered. ICRS macro scores showed significant differences between the NSSIS + BMSCs group and NSSIS group, with both groups scoring higher than the control group (*p* < 0.05, Fig. 1). The cartilage surface morphology scores in the NSSIS + BMSCs group were significantly higher than those in NSSIS group (*p* < 0.01) and the control group (*p* < 0.05, Fig. 1). At 48 h, the NSSIS + BMSCs group also showed significantly better scores than the control group (*p* < 0.05, Fig. 1).

### Histological observation of NSSIS and sustained release of growth factors

**Fig. 2:**
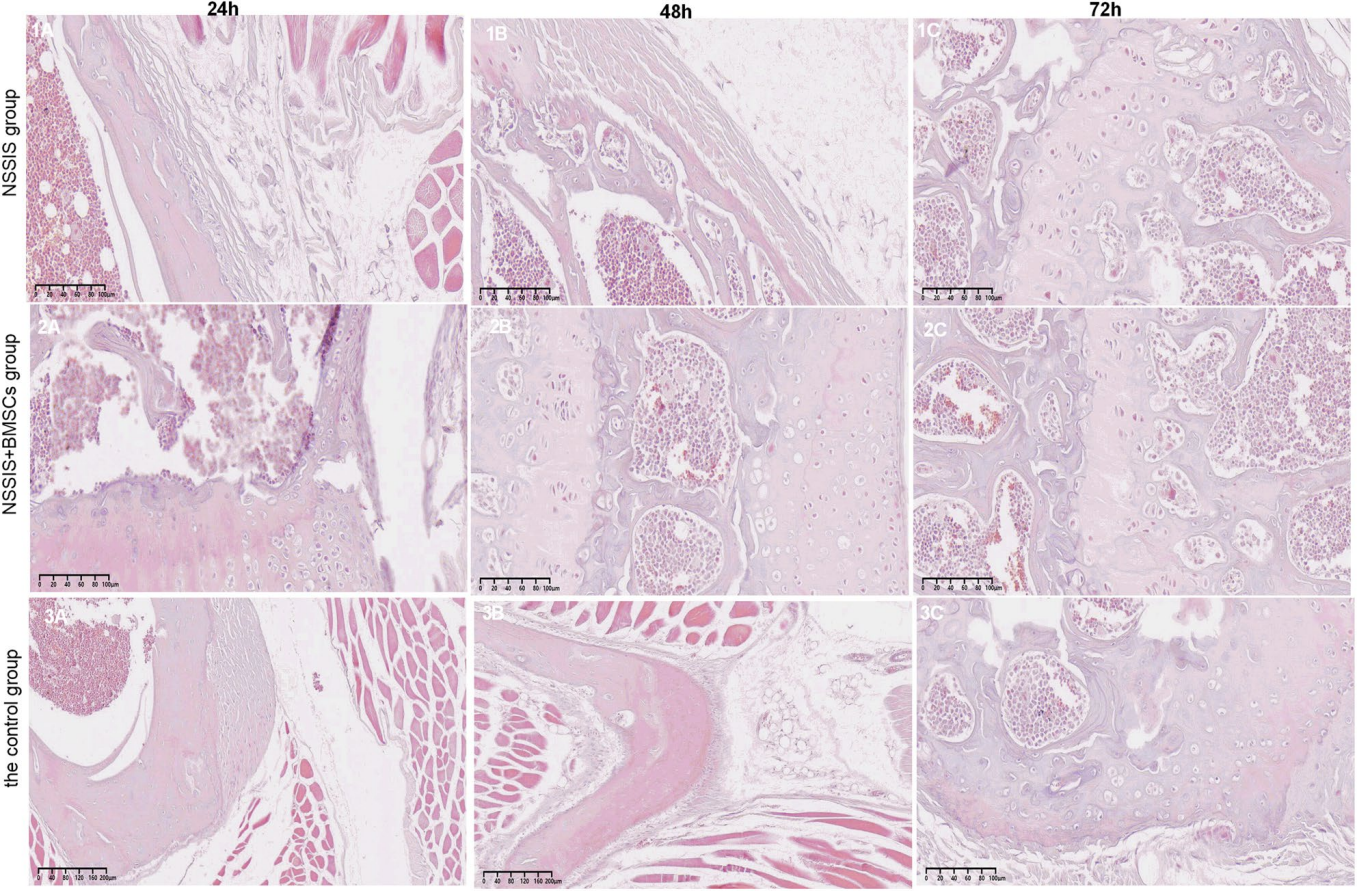
Histological observation of rabbit BMSCs after implantation of NSIS in vitro

Live/Dead Cell Staining: Most BMSCs were stained green, indicating viability, with only a few red-stained dead cells (Fig. 2). These findings suggests that NSSIS is non-cytotoxicity and biocompatible with rabbit BMSCs.

Histological Features of NSSIS: H&E staining showed a sparse fibrous network capturing leukocytes and platelets within the matrix (Fig. 2). The loose structure appears favorable for cell adhesion, growth, and nutrient transport, supporting its potential as a scaffold material for tissue engineering.

**Fig. 3:**
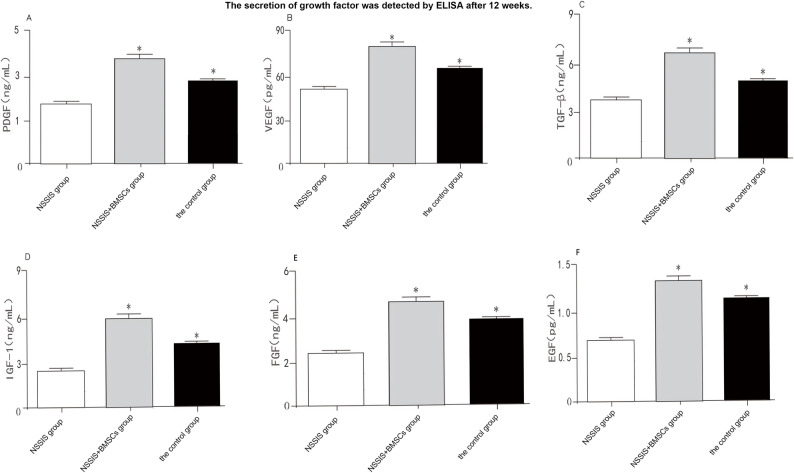
Influence of cartilage tissue factor content in three groups of rabbits (x̄ ± s, *n* = 12) Note: *p < 0.05 indicates a significant difference in the NSSIS group compared to the control group

Growth Factor Release Profile: ELISA revealed sustained release of PDGF, VEGF, TGF-β, insulin-like growth factor 1 (IGF-1), FGF, and EGF (Fig. 3), indicating that NSSIS may promote cell proliferation and differentiation through these growth factors.

### The result of histological staining observation

#### The result of hematoxylin-eosin (H&E) staining

**Fig. 4:**
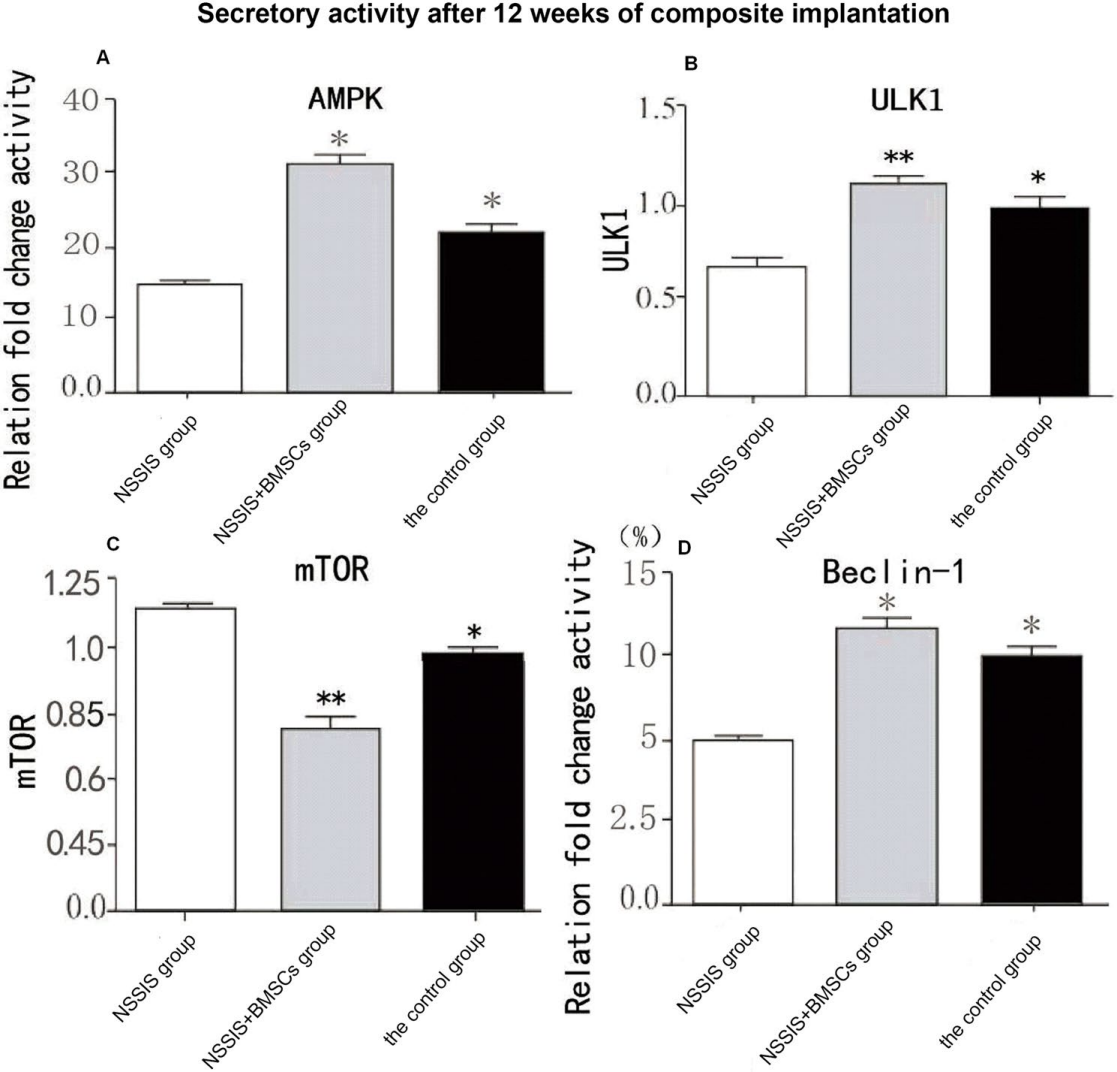
Microscopic observation of three groups of cartilage tissues (hematoxylin-eosin staining × 200)

In the NSSIS group, the growth plate cartilage was interrupted and the repair tissue appeared disordered. In contrast, the NSSIS + BMSCs group showed repair cartilage arranged in a column-like pattern within the growth plate area. In the control group, no repair was observed, and cartilage discontinuity persisted at 72 h after implantation (Fig. 4).

Histologically, the NSSIS group displayed few chondrocytes, with subchondral bone and trabeculae partially broken and gradually replaced by loose fibrous connective tissue. In the NSSIS + BMSCs group, new chondroid cells gradually grew into the defect edge. These cells were smaller and sparser than those in surrounding normal tissue, but partial continuity between the subchondral bone and adjacent cartilage was observed. Cell arrangement gradually became more orderly, with a subset of cells showing maturation. In contrast, the control group showed complete degeneration of chondrocytes, loss of the subchondral bone calcification layer, and disappearance of trabeculae (Fig. 4).

Overall, NSSIS served as an effective filler, supporting growth plate repair and cartilage regeneration. Its loose histological structure appears favorable for stromal cell adhesion, migration, and nutrient transport, underscoring its potential as a scaffold material.

#### The result of Alizarin red and Alizarin red hematoxylin staining

**Fig. 5:**
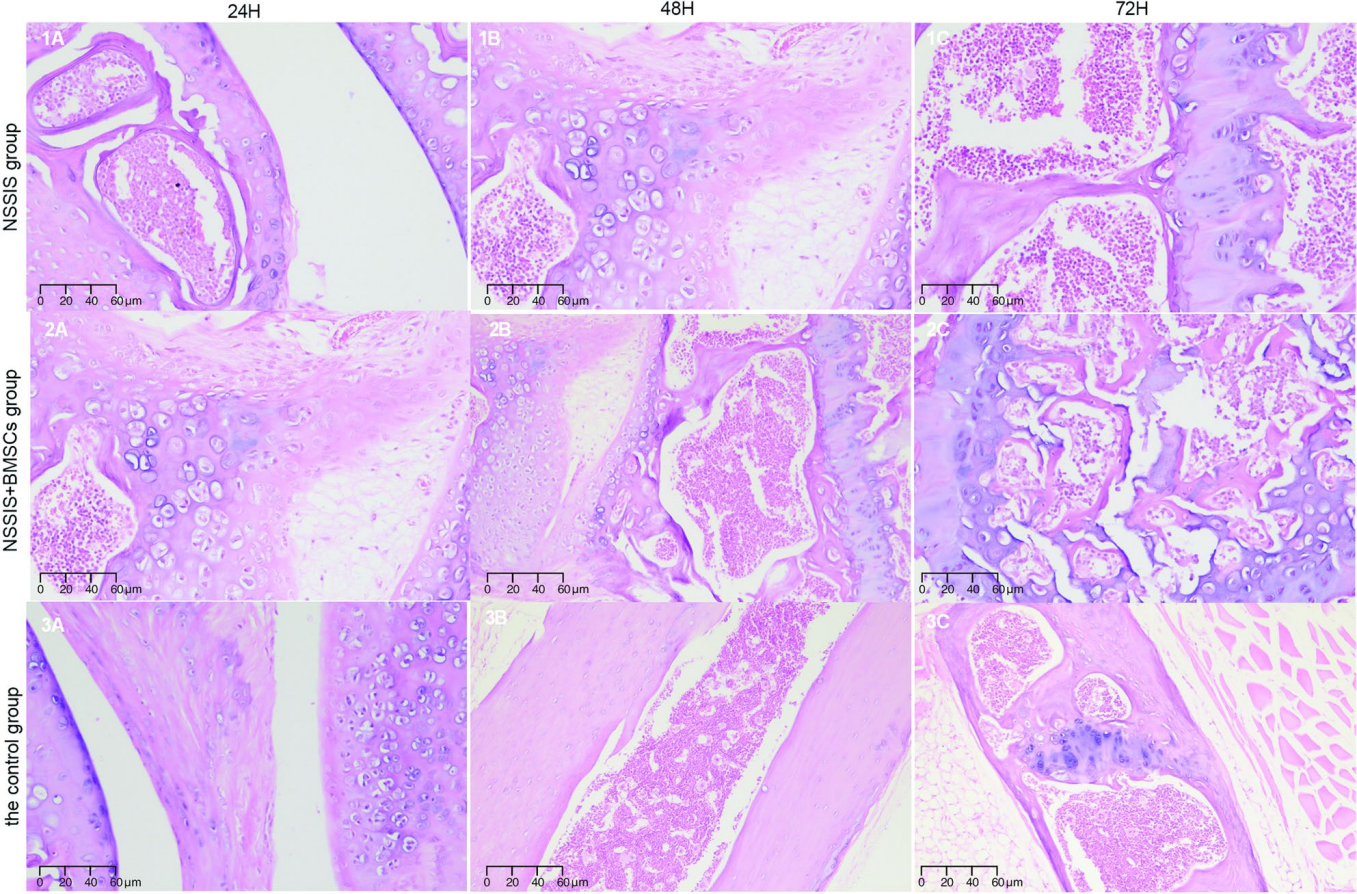
Microscopic observation of three groups of cartilage tissues (alizarin red hematoxylin staining × 200)

In both the NSSIS and NSSIS + BMSCs groups, cartilage defects were largely filled with new tissue. The repair margins, of “critical ridge”, contained pink-orange, fusiform collagen fibers (Fig. 5). However, metachromatic cartilage matrix formation was limited in the NSSIS group and modest in the BMSCs group.

In the NSSIS + BMSCs group, subchondral bone defects were filled with new bone tissue and showed greater metachromatic cartilage matrix formation. The implants integrated well with surrounding cartilage and subchondral bone, without a fibrous tissue interface (Fig. 4). In contrast, the control group showed no stained cartilage matrix and no new tissue filling the defect.

Compared with NSSIS alone, the NSSIS + BMSCs group demonstrated superior repair. The regenerated cartilage and subchondral bone layers were similar in thickness to adjacent primary cartilage, and the mature trabecular structure near the scaffold resembled that of normal chondrocytes (Fig. 5).

#### The result of toluidine blue staining

**Fig. 6:**
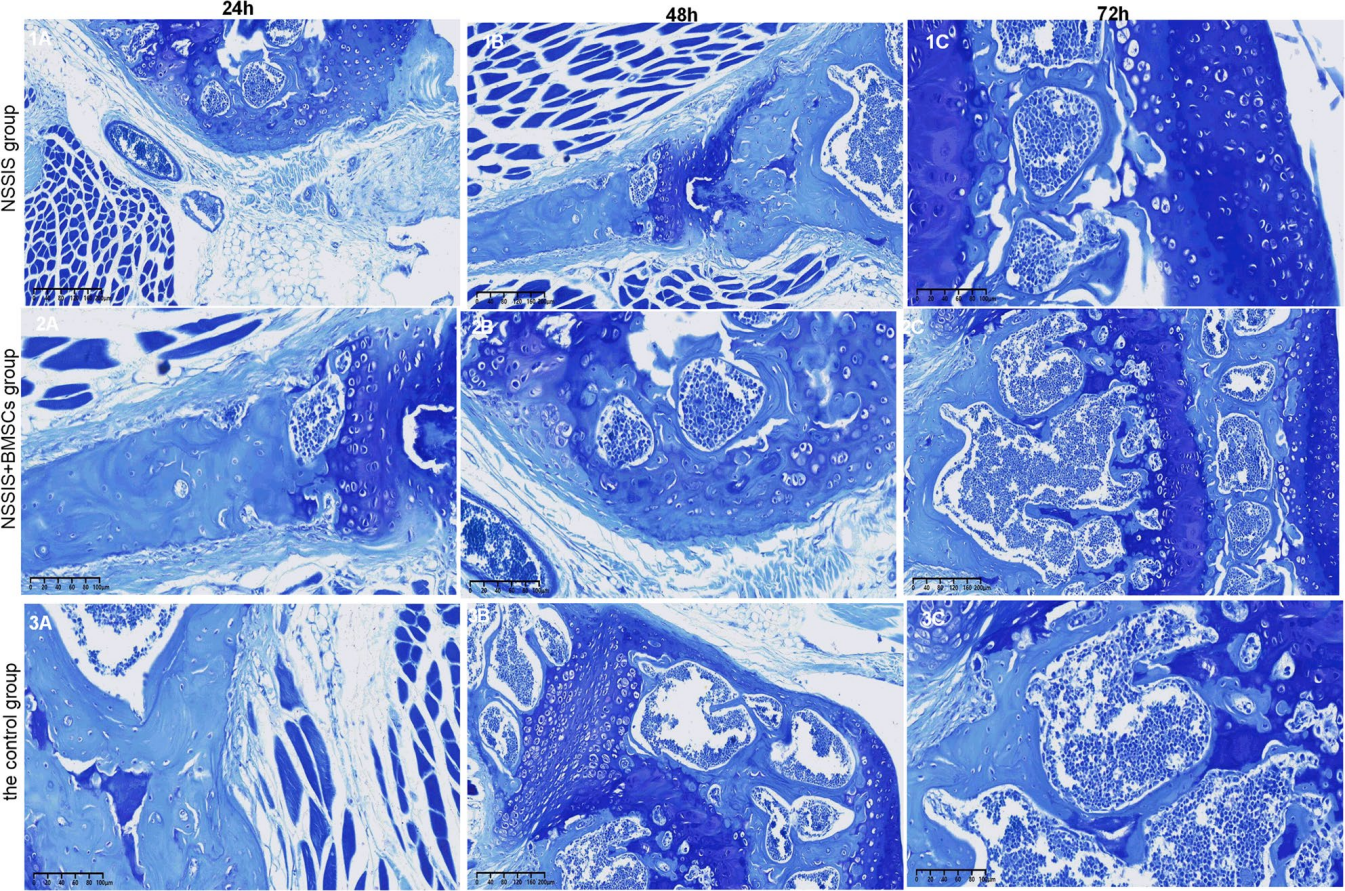
Microscopic observation of three groups of cartilage tissues (toluidine blue staining × 200)

In the NSSIS group, new cartilage tissue contained relatively less purplish-blue glycosaminoglycan, reflecting reduced polysaccharide binding (Fig. 6). In contrast, the NSSIS + BMSCs group showed more distinct chondrocytes and abundant purplish-blue acid mucosaccharides such as chondroitin sulfate, while surrounding tissues stained light blue. The new cartilage layer was significantly thicker than in the NSSIS group (mean thickness score: 3.8 ± 0.5 vs. 2.1 ± 0.4, *p* < 0.05), and the fibropericharpium at the surface appeared more developed. Safranin O staining intensity was also higher in the NSSIS + BMSCs group (mean staining score: 3.6 ± 0.3 vs. 2.0 ± 0.4, *p* < 0.05), indicating increased glycosaminoglycan content and better cartilage matrix restoration.

However, the reconstructed cartilage and subchondral bone were still immature, and the tidal line remained indistinct. In the control group, the defect area lacked new cartilage or bone coverage and was only filled with fibrous tissue (Fig. 6). No obvious glycosaminoglycan staining was detected in this group. The NSSIS scaffold appeared to provide a favorable environment for cartilage repair. Its porous, reticular structure supported the retention of cells, platelets, and growth factors, promoting cell proliferation, differentiation, and nutrient diffusion. The presence of morphologically immature cartilage cells in the repaired tissue suggests ongoing remodeling, consistent with the relatively short observation period.

### Gene expression of BMSCs

**Fig. 7:**
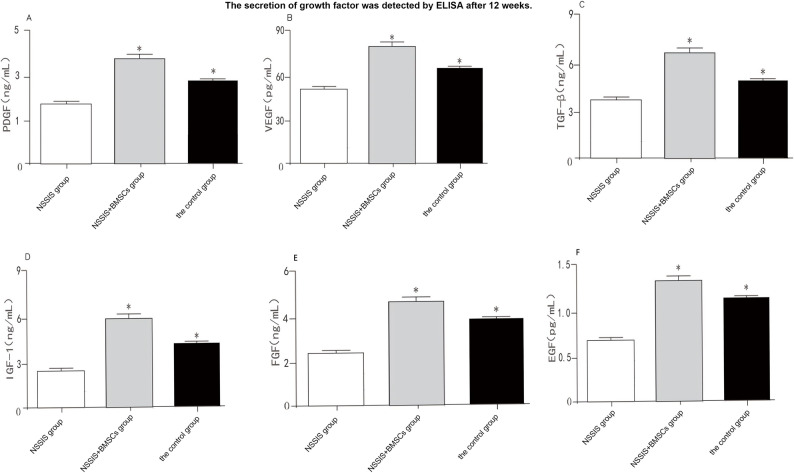
Comparison of receptor expression levels in rabbits’ cartilage tissues among of three groups (x̄ ± s, *n* = 12) Note: *p < 0.05 indicates a significant difference in NSSIS group compared to the control group; **p < 0.05 indicates a significant difference in NSSIS group compared to NSSIS + BMSCs group

The protein and mRNA expression levels of AMPK, ULK1, and Beclin-1 were significantly higher in the NSSIS group compared to the control, while mTOR expression was significantly lower (*p* < 0.05, Fig. 7). These markers were further elevated in the NSSIS + BMSCs group compared with the NSSIS group (*p* < 0.05), whereas mTOR expression was decreased.

Compared with NSSIS + BMSCs, the control and NSSIS groups showed significantly lower AMPK, ULK1, and Beclin-1 mRNA levels and higher mTOR mRNA expression (*p* < 0.01). Conversely, the NSSIS + BMSCs group exhibited significantly increased AMPK, ULK1, and Beclin-1 mRNA and decreased mTOR mRNA relative to the control group (*p* < 0.05). These results indicate that the combination of NSSIS with BMSCs further enhances autophagy-related signaling in cartilage tissue compared with NSSIS alone or control treatment.

### Comparison of protein expressions of AMPK, mTOR, ULK1 and Beclin-1 in knee cartilage tissue of rabbits in each group after intervention

**Fig. 8:**
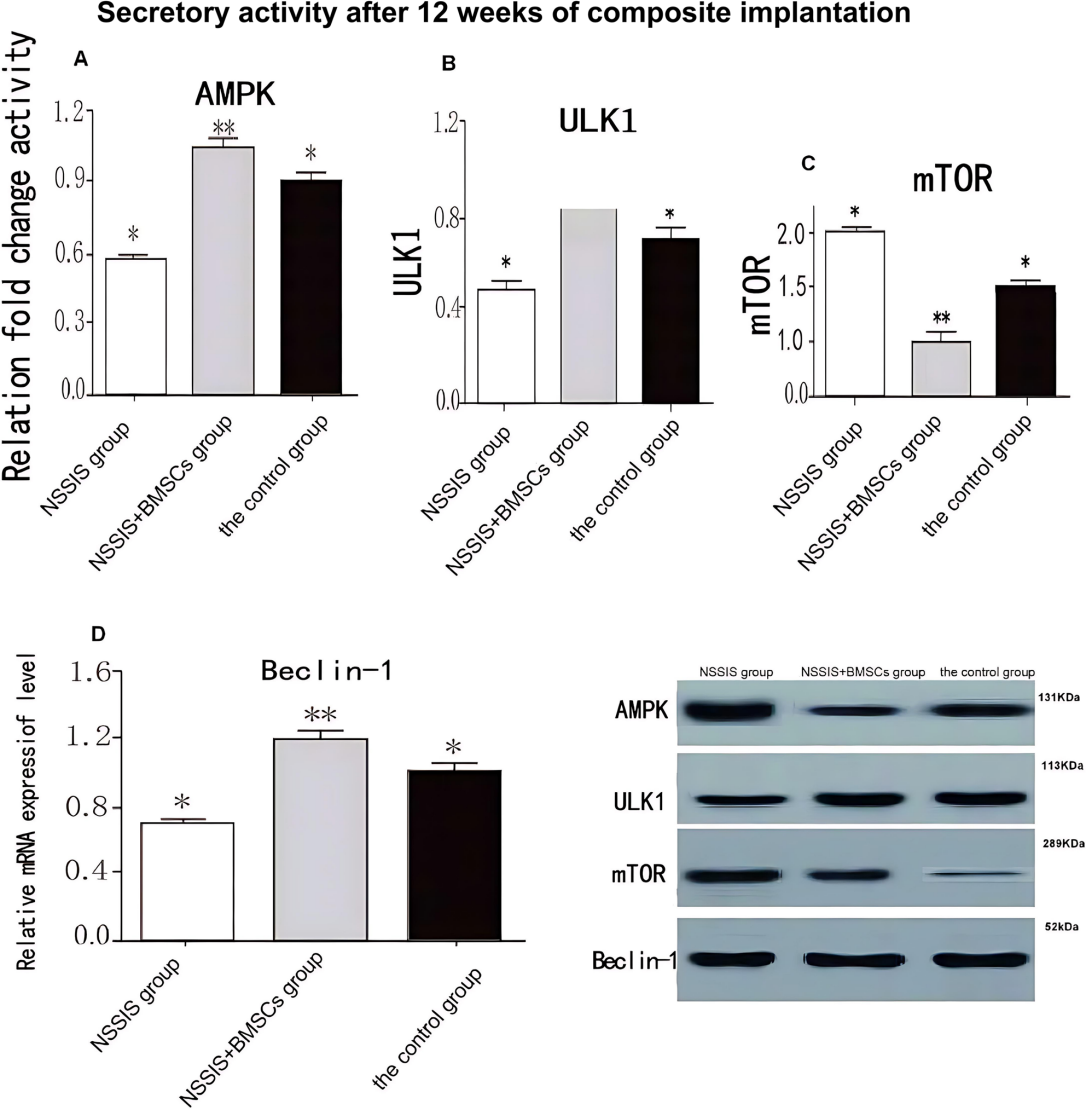
Comparison of mRNA expression levels of AMPK, mTOR, ULK1 and Beclin-1 in knee cartilage of three groups rabbits (x̄ ± s, *n* = 12) Note: *p < 0.05 indicates a significant difference in the NSSIS group compared to the control group; **p < 0.05 indicates a significant difference in the NSSIS + BMSCs group compared to the NSSIS group

In rabbit cartilage, mRNA expressions of AMPK, ULK1, and Beclin-1 were significantly higher in the NSSIS + BMSCs group compared with the NSSIS group, while mTOR mRNA was significantly lower (*p* < 0.01). Compared with the control group, the NSSIS group showed significantly increased AMPK, ULK1, and Beclin-1 expression and decreased mTOR protein levels (*p* < 0.05, Fig. 8). The NSSIS + BMSCs group exhibited the highest expression of AMPK, ULK1, and Beclin-1 and the lowest mTOR expression relative to the control (*p* < 0.01, Fig. 8). Additionally, the repair tissue in the NSSIS + BMSCs group contained more newly synthesized proteins than normal cartilage, likely reflecting ongoing cartilage regeneration and the immature state of newly formed tissue at this postoperative time point.

## Discussion

Cartilage around joints is essential for bearing mechanical loads and enabling smooth movement. Defects in cartilage can cause severe pain and mobility limitations. A major challenge in cartilage tissue engineering is ensuring that regenerated tissue integrates structurally and functionally with native cartilage [26–28]. Poor integration between new and native matrix is often a primary reason for repair failure.

In this study, the NSSIS + BMSCs group achieved the highest ICRS scores and showed superior cartilage repair. Compared with the NSSIS group, cartilage coverage was thicker. In the knee defect model, NSSIS alone nearly completely filled the defect, and the regenerated cartilage integrated well with surrounding tissue. No obvious fibrous interface was observed, indicating strong structural and functional repair. Previous studies using SIS, such as Dontu et al. [29] and Li et al. [30], also demonstrated effective cartilage repair and stem cell proliferation, supporting the utility of SIS-based scaffolds. Nanotechnology, with its profound ability to manipulate matter at the atomic and molecular scale, has heralded a new epoch of scientific and technological advancement, promising revolutionary solutions across diverse sectors including medicine, electronics, energy, and environmental remediation. The synthesis of a wide variety of nanomaterials, via green pathways, includes metallic (Ag, Au, Cu, Pt, Pd), metal oxide (ZnO, Fe-oxides, TiO2), and chalcogenide (Se, QDs) nanoparticles.

In vitro, H&E staining of NSSIS revealed a sparse fibrous structure conducive to cell adhesion, growth, and nutrient transport. Produced via low-speed, long-term centrifugation, NSSIS provides a porous, fibrous protein scaffold that carries cells, platelets, and growth factors while promoting oxygen and nutrient diffusion. This structure ensures stable retention of biological factors and maintains scaffold integrity in PBS for at least two months [31, 32], matching the degradation rate to cartilage regeneration and providing continuous support during repair. Compared with SIS, NSSIS offers improved structural and functional properties for cartilage tissue engineering.

ELISA analysis of key secreted growth factors such as PDGF, VEGF, TGF - β, IGF-1, FGF, and EGF showed sustained release, confirming that NSSIS has good biocompatibility and can promote the chondrocyte growth trend of BMSCs, which is very similar to the biological characteristics of cartilage proliferation and subchondral bone regeneration and repair [33]. It has the potential for chondrogenesis [34] and supports mechanical stability and nutrient diffusion. When applied to cartilage defects, the scaffold can effectively interact with the surrounding natural cartilage and subchondral bone. Cartilage lacks vascular antigenicity and often undergoes degeneration or ossification after transplantation. The regulation of growth factors TGF-β, IGF-1, and FGF has a significant promoting effect on the synthesis of DNA [35], collagen, and proteoglycans in chondrocytes, PDGF, Capture the expression of phenotype genes in chondrocytes under the action of IGF-1, while VEGF inhibits the metabolism of chondrocytes [36]. The interactive and combined application of six factors shows a dose-response relationship and plays an important role in cartilage regeneration and repair. From this experiment, it can be concluded that NSSIS promotes the sustained release of growth factors through the natural degradation of fibrin. The best clinical application is in cartilage injuries with a thickness greater than 50% of cartilage. However, this conclusion is only based on the experimental results of rabbits. Before it can be applied in clinical practice, it still requires a large number of experiments using human-derived cells and in vivo experiments for verification. Nevertheless, we believe that this conclusion provides a promising option for future treatment plans.

After implantation, we observation by H&E staining showed that NSSIS + BMSCs group had more repair cartilage the length and width of regenerated cartilage increasing in a columnar manner in the intercondylar fossa defect repair area than the NSSIS group. Strong alkaline cartilage pits with irregular cavities were observed on the glass slides, and BMSCs could be induced to differentiate into chondrocytes under appropriate induction conditions; Compared with NSSIS group, which only had a small amount of connective tissue between the repaired cartilage, the control group had interrupted growth layer of cartilage plates, confirming that NSSIS has significant advantages in promoting angiogenesis and cartilage tissue regeneration, with strong self-renewal ability and multi-directional differentiation potential.

Alizarin Red–Hematoxylin staining showed that implants and subchondral bone defects gradually formed a dense, heterochromatic cartilage matrix in the NSSIS + BMSCs group, with no heteromorphic bands compared to the other groups. This indicates ongoing scaffold degradation, continued cell proliferation, and cartilage matrix secretion, ultimately supporting living cartilage formation. Toluidine blue staining revealed that the new cartilage layer in the NSSIS + BMSCs group was significantly thicker than in the NSSIS group, with abundant purple-blue acidic mucopolysaccharides, confirming that NSSIS serves as a degradable scaffold, gradually disappearing over time while supporting BMSC proliferation and differentiation into functional chondrocytes [37]. This process promotes cartilage protein regeneration, inhibits degradation, and helps restore joint structure and function.

After large-scale expansion, BMSCs were seeded onto chondrocytes capable of synthesizing matrix and fibers, enhancing cartilage elasticity and maintaining joint load-bearing and buffering functions.[38] Previous studies comparing SIS and NSSIS in rabbit osteochondral defects demonstrated that both scaffolds support chondrocyte proliferation and tissue repair; however, NSSIS showed superior early-stage proliferation and tissue regeneration, making it a promising choice for osteochondral repair [39, 40]. The scaffold’s porous, three-dimensional structure enriches cells, neutrophils, and platelets, supporting angiogenesis and soft and hard tissue regeneration [41]. Notably, NSSIS induces cartilage regeneration without artificial reagents, minimizing systemic or local immune reactions, and providing a favorable environment for sustained tissue repair.

We found that BMSCs treated with NSSIS were activated toward cartilage-specific gene expression. qRT-PCR analysis revealed significant upregulation of AMPK, ULK1, and Beclin-1 mRNA, while mTOR expression was significantly decreased. Western blot analysis confirmed increased AMPK and ULK1 protein levels, indicating active growth of regenerated cartilage. The transient increase in Beclin-1 may reflect endochondral ossification, promoting chondrocyte differentiation and maturation, while downregulation of mTOR impacts chondrocyte metabolism [42]. Activation of macrophages to synthesize AMPK, ULK1, and Beclin-1 may enhance cartilage regeneration through pro-inflammatory cytokine signaling, promoting endothelial repair and chondrocyte activity. ULK1 and Beclin-1 further support extracellular matrix and fibrochondroitin sulfate synthesis. During vascular repair, mTOR does not directly act on endothelial cells, allowing chondrocytes in epiphyses to maintain matrix formation and attract multinucleated giant cells, establishing a positive balance of chondrocyte growth factors [42]. 

qRT-PCR analysis indicated that AMPK and ULK1 significantly induce Beclin-1 expression, further supporting chondrogenic differentiation of rabbit BMSCs. The observed activation of the ULK1/Beclin-1/mTOR axis confirms that NSSIS promotes differentiation and proliferation of BMSCs into chondrocytes. Upregulation of this axis marks the initiation of cartilage regeneration, with gradual maturation of secreted matrix and fibers, entering early chondrogenesis for repair.

These findings suggest that NSSIS may also facilitate the homing of mesenchymal stem cells, potentially enhancing recruitment of endogenous cells for tissue repair. Compared with Tao et al., [43] this study demonstrates the direct effect of NSSIS on BMSC-mediated cartilage regeneration, confirming the pivotal role of BMSCs in initiating cartilage repair. Meanwhile, the fiber structure of NSSIS plays a crucial role as a scaffold. AMPK can sensitively sense changes in the intracellular AMP/ATP secretion ratio, thereby rapidly activating downstream pathways. Regulate the uptake, oxidation, and mitochondrial biosynthesis of sugars and fats within the gap between muscle bone cartilage cells, enabling rapid infiltration into the cartilage matrix. NSSIS effectively promotes the migration of rabbit BMSCs and has a more pronounced chemotactic effect on BMSCs compared to surrounding chondrocytes.

After the implantation of NSSIS + BMSCs complex, ULK1 undergoes phosphorylation and activates AMPK. The activated AMPK forms a signal transduction cascade, inhibiting the binding of factors to mTOR release subunits. Therefore, a large amount of AMPK signal transduction promotes mitochondrial aggregation in microcapillary walls, reduces autophagosomes, increases fibrocartilage components in the extracellular matrix of chondrocytes, expands the rough endoplasmic reticulum, and enhances the biocompatibility of NSSIS + BMSCs with proteoglycans and collagen fibers in the cartilage matrix. With the increase of local secretion of Beclin-1, the nucleus grows from crescent to large vesicles, and chromatin aggregates in the inner layer of the nuclear membrane. Under transmission electron microscopy, the elastic protein in the extracellular matrix of chondrocytes increases significantly, and the regenerated soft lattice surface tends to be smooth; Although there are no blood vessels in the cartilage, the vascular wall ridges in the non-cartilage area of the NSSIS + BMSCs binding interface at the site of injury in the model rabbit elongate and neovascularization increases, which may be related to its mediation of the AMPK-mTOR-ULK1 signaling pathway.

The advantages and limitations of NSSIS in cartilage repair in this study coexist. Silver has been widely used as a topical antimicrobial agent, especially in repairing contaminated wound. NSSIS could provide a natural microenvironment conducive to cell growth and have broad prospects for applications in regenerative medicine and osteochondral tissue engineering. NSSIS precise and controllable prosthetic cartilage transplantation, speculatively in vitro monolayer culture proliferation, cross-linking between fibrin and matrix can enhance its mechanical integrity and slow down its degradation [44]. However, the degree of degradation control and immune rejection are also the main factors restricting transplantation.

## Conclusions

The research results indicate that NSSIS effectively enhances the expression of chondrocyte related genes in BMSCs, and their secretion and deposition are crucial for cartilage repair and regeneration. NSSIS significantly promoted the in situ chondrogenic differentiation of rabbit BMSCs, and enhanced the migration of BMSCs and chondrocytes, confirming that BMSCs have the potential for cell recruitment in vivo and serve as a starter for tissue regeneration.

## Data Availability

The datasets used and/or analyzed during the current study are available from the corresponding author upon reasonable request.

## Abbreviations

NSSIS: Nano silver small intestine submucosa
BMSCs: bone marrow mesenchymal stem cells
PDGF: platelet-derived growth factor
VEGF: vascular endothelial growth factor
TGF-β: Transforming growth factor beta
IGF-1: Insulin-like growth factor 1
FGF: fibroblast growth factor
EGF: Epidermal Growth Factor
AMPK: Adenosine 5-monophosphate (AMP)-activated protein kinase
mTOR: mammaliantargetofrapamycin
ULK1: Unc-51-like kinase 1
Beclin 1: Beclin 1 protein
PAA: Polyacrylic Acid
MPss: Methylprednisolone sodium succinate
H&E: hematoxylin-eosin staining
ELISA: enzyme linked immunosorbent assay
PRF: Platelet rich fibrin
ALP: Alkaline Phosphatase
